# Intraoperative development of pulmonary thromboembolism in a bedridden patient owing to a pelvic bone fracture with negative preoperative computed tomography pulmonary angiographic findings: A case report

**DOI:** 10.1097/MD.0000000000026658

**Published:** 2021-07-23

**Authors:** Jong Hae Kim, Hyungseop Lim, Hyun Mi Kim, Jung A. Lim

**Affiliations:** a Department of Anesthesiology and Pain Medicine, School of Medicine, Daegu Catholic University; b Department of Obstetrics and Gynecology, School of Medicine, Kyungpook National University, Daegu, Korea.

**Keywords:** computed tomographic pulmonary angiography, pelvic bone fracture, pulmonary thromboembolism, tachycardia

## Abstract

**Rationale:**

Pulmonary thromboembolism (PTE) is a potentially life-threatening condition with high morbidity and mortality, and computed tomographic pulmonary angiography (CTPA) is an important diagnostic tool for patients in whom PTE is suspected; however, intraoperative PTE is very difficult to diagnose and often has a rapid clinical course. We experienced a case of intraoperative PTE with persistent tachycardia refractory to conventional treatments despite negative preoperative CTPA findings.

**Patient concerns:**

A 53-year-old man with a pelvic bone fracture who had been on bed rest for 10 days underwent open reduction and internal fixation under general anesthesia. He remained tachycardic (heart rate of 120 beats/min) despite treatments with fluid resuscitation, analgesics, and beta-blockers.

**Diagnoses:**

Preoperative CTPA, computed tomography (CT) venography, and transthoracic echocardiography showed no signs of deep vein thrombosis and PTE. However, the levels of D-dimer were elevated. After the start of the surgery, tachycardia (heart rate between 100 and 110 beats/min) could not be treated with fluid resuscitation. Systolic blood pressure was maintained between 90 and 100 mm Hg using continuous infusion of phenylephrine. Ninety minutes after the surgery, systolic and diastolic blood pressures suddenly dropped from 100/60 to 30/15 mm Hg with a decrease in end-tidal carbon dioxide concentration from 29 to 13 mm Hg and development of atrial fibrillation. Arterial blood gas analysis revealed hypercapnia. Under the suspicion of PTE, cardiopulmonary resuscitation (CPR) was immediately initiated. Three CPR cycles raised the blood pressure back to 90/50 mm Hg with sinus tachycardia (115 beats/min). Transesophageal echocardiography showed right ventricular dysfunction and paradoxical septal motion. However, emboli were not found. Postoperative chest CT revealed massive PTE in both pulmonary arteries.

**Interventions:**

Immediately, surgical embolectomy was performed uneventfully.

**Outcomes:**

The patient was discharged from the hospital 1 month later without any complications.

**Lessons:**

The patient with moderate risk for PTE (heart rate > 95 beats/min and immobilization, surgery under general anesthesia, and lower limb fracture within 1 month) should be closely monitored and managed intraoperatively even if preoperative CTPA findings are negative. The development of PTE needs to be expected if tachycardia is refractory to conventional treatments.

## 1 Introduction

Pulmonary thromboembolism (PTE) is a rare but potentially life-threatening condition associated with high morbidity and mortality.^[1,2]^ The prevalence of PTE is 4.6% in critically ill trauma patients (n = 153) receiving thromboprophylaxis therapy, among whom patients with pelvic bone fracture had an odds ratio of 3.04 for the development of venous thromboembolism.^[3]^ In addition, the incidence of PTE was the highest (0.78%) in patients undergoing pelvic open reduction and internal fixation among 11,313 adult trauma patients on thromboprophylaxis therapy.^[4]^ Therefore, more attention should be paid to the development of PTE in patients with a pelvic bone fracture.

Perioperatively (before and after surgery), computed tomographic pulmonary angiography (CTPA) and D-dimer test are the mainstay for diagnosing PTE in patients with suspected PTE in the absence of hemodynamic instability.^[5]^ However, intraoperative PTE is very difficult to diagnose and often has a rapid clinical course. Furthermore, the results of preoperative tests do not take effect until the intraoperative period because the physiological milieu changes under both the anesthetic and surgical conditions. Nevertheless, there are limited predictive factors for PTE and no available guidelines for its screening. The obstruction of the pulmonary arteries (the mechanism of PTE) is often accompanied by nonspecific hemodynamic changes, such as normal blood pressure compensated for by tachycardia.^[6]^ Catecholamine-driven tachycardia in combination with right ventricular dilatation is an important compensatory mechanism to maintain cardiac output. However, normal blood pressure with tachycardia may not always indicate PTE because other confounding factors affect intraoperative hemodynamics, such as underlying cardiovascular or autonomic diseases, anesthetics, bleeding, fluid shifting, etc. Therefore, it would be reasonable to suspect the occurrence of PTE in patients at risk for PTE if tachycardia persists even after all the candidate causes other than PTE are ruled out. In addition, given the fact that the development of shock and the severity of hemodynamic compromise are important predictive and prognostic factors for mortality,^[7]^ close monitoring of vital signs to identify and halt the progress of PTE is mandatory.

Herein, we report intraoperative development of PTE preceded by tachycardia refractory to conventional treatments in a bedridden patient owing to a pelvic bone fracture with no evidence of deep vein thrombosis (DVT) and PTE on preoperative CTPA and computed tomography (CT) venography.

## 2 Case report

A 53-year-old man (height, 180 cm; weight, 76.6 kg), a heavy alcoholic, was admitted to a local medical center under the diagnosis of right acetabular and iliac wing fracture after falling from a tree. The patient was bedridden under conservative treatment. Three days later, he presented with abdominal pain and tenderness and was then transferred to our hospital for further evaluation and management. The systolic/diastolic blood pressure, heart rate on electrocardiogram, and body temperature were 160/94 mm Hg, 120 beats/min, and 37.5°C, respectively. Under the suspicion of dehydration and fracture-induced pain, fluid resuscitation was initiated with Hartmann solution and analgesics were administered. Nonetheless, the heart rate did not decrease. Hence, bisoprolol (5 mg per os) was administered once daily. Thiamine, haloperidol, and quetiapine were administered to treat alcohol withdrawal delirium. Every time the patient became responsive to verbal stimuli, he kept reporting abdominal pain. However, no pulmonary symptoms were present. Anteroposterior chest radiograph and transthoracic echocardiography (TTE) were normal. Abdominal CT revealed retroperitoneal and right iliacus hematoma. However, no injury was found in major vessels or organs. The laboratory findings showed increased levels of D-dimer (26.64 μg/mL), the normal range of which was < 0.1 μg/mL. Because he was immobilized after the injury with elevated levels of D-dimer, combined CT venography and CTPA was performed 7 days after the injury to rule out DVT and PTE; however, the results were negative.

Three days later (10 days after the injury), he presented to the operating room for open reduction and internal fixation of the pelvic bone fracture. Before anesthesia induction, the systolic/diastolic blood pressure, heart rate, and peripheral oxygen saturation (SpO_2_) were 142/92 mm Hg, 100 beats/min, and 100%, respectively. Anesthesia was induced with bolus administration of 130 mg propofol and continuous infusion of remifentanil at 0.1 μg/kg/min. Endotracheal intubation was facilitated with 50 mg rocuronium. The ventilator was set in the volume-controlled mode to deliver a tidal volume of 6 to 8 mL/kg at a respiratory rate of 10 to 12 breaths/min. The tidal volume and respiratory rate were adjusted to maintain end-tidal carbon dioxide concentration (EtCO_2_) between 33 and 35 mm Hg. Anesthesia was maintained with sevoflurane (1.5–2.0 vol%) in oxygen/air mixture (inspired oxygen saturation of 50%) to ensure bispectral index values between 40 and 60. The infusion rate of remifentanil (0.1–0.2 μg/kg/min) was adjusted to maintain blood pressure and heart rate within 20% of the baseline values. The right radial artery was catheterized for real-time monitoring of arterial blood pressure and arterial blood sampling. The right internal jugular vein was catheterized for real-time monitoring of central venous pressure, bolus administration of drugs, and continuous infusion of drugs. The results of arterial blood gas analysis after anesthesia induction were as follows: pH, 7.468; partial pressure of arterial carbon dioxide (PaCO_2_), 37.2 mm Hg; partial pressure of arterial oxygen (PaO_2_), 193.5 mm Hg; bicarbonate (HCO_3_^−^), 26.3 mmol/L; base excess, 2.6 mmol/L; hemoglobin, 8.2 g/dL.

**Figure 1:**
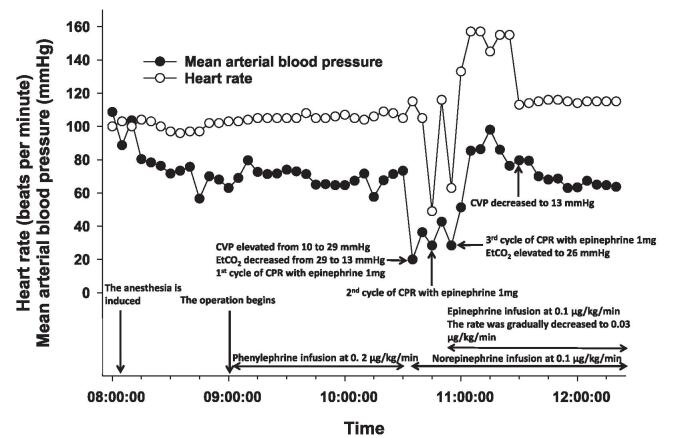
Anesthesia record. CPR = cardiopulmonary resuscitation, CVP = central venous pressure, EtCO_2_ = end-tidal concentration of carbon dioxide.

At 10 minutes before the surgery, 100 μg phenylephrine was bolus administered twice at a 5-minute interval to ensure a systolic blood pressure of > 100 mm Hg. At the beginning of the surgery, phenylephrine was continuously infused at a rate of 0.2 μg/kg/min owing to the transient effects of its bolus administration. To compensate for intraoperative blood loss and preoperative dehydration, 6% hydroxyethyl starch, packed red blood cells, and fresh frozen plasma were administered at a rate of 8 to 10 mL/kg/min, and systolic blood pressure and heart rate were maintained between 90 and 100 mm Hg and between 100 and 110 beats/min, respectively. Ninety minutes after the surgery, systolic/diastolic arterial blood pressure and EtCO_2_ abruptly decreased from 100/60 to 30/15 mm Hg and from 29 to 13 mm Hg, respectively. The central venous pressure elevated from 10 to 29 mm Hg. The heart rate slightly increased from 105 to 115 beats/min. The normal sinus rhythm on the electrocardiogram turned into atrial fibrillation, after which bradycardia (49 beats/min) ensued. Under the impression of PTE, 3 cycles of cardiopulmonary resuscitation (CPR) were intermittently performed for 25 minutes until spontaneous circulation [systolic/diastolic blood pressure of 80–90/50 mm Hg and sinus tachycardia (115 beats/min)] was achieved (Fig. 1). At the beginning of CPR, epinephrine and norepinephrine were infused at a rate of 0.1 μg/kg/min, and 3 mg epinephrine was bolus administered during CPR. Arterial blood gas analysis performed between the first 2 CPR cycles revealed hypercapnia (pH, 7.287; PaCO_2_, 55.8 mm Hg; PaO_2_, 98.7 mm Hg; HCO_3_^−^, 26.0 mmol/L; base excess, −0.6 mmol/L; hemoglobin, 8.8 g/dL). At the end of the last CPR cycle, the infusion rate of the fluid was reduced to 3 to 5 mL/kg/min, and a probe for transesophageal echocardiography (TEE) was inserted into the esophagus. TEE showed right ventricular dysfunction with paradoxical septal motion. However, no embolus was found in the main, right, and left pulmonary arteries. Intraoperatively, 500 mL of 6% hydroxyethyl starch, 1250 mL of packed red blood cells, and 450 mL of fresh frozen plasma were administered in total. The amount of blood loss and urine output was 700 and 100 mL, respectively.

**Figure 2:**
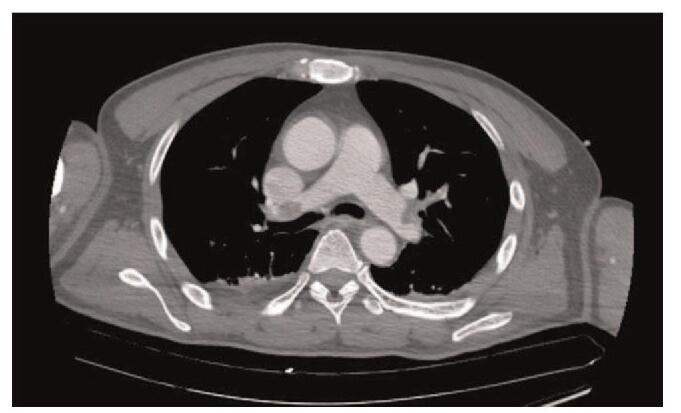
Chest computed tomography showing the emboli in both pulmonary arteries.

**Figure 3:**
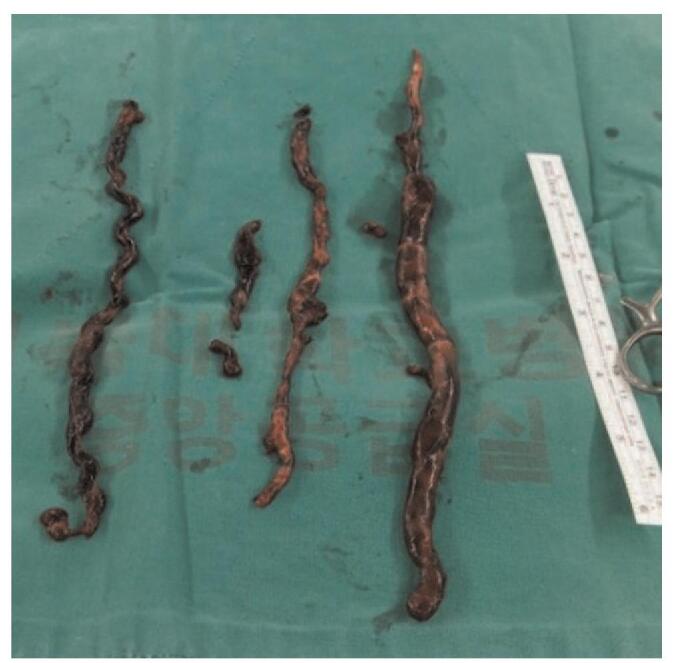
Emboli retrieved from both pulmonary arteries.

Chest CT performed immediately after the surgery showed emboli in both the pulmonary arteries (Fig. 2). The patient was taken back to the operating room and underwent surgical embolectomy under cardiopulmonary bypass with aortic cross-clamping and cardioplegic arrest. The incision was made in both the main pulmonary arteries, and emboli were removed (Fig. 3). EtCO_2_ and central venous pressure were elevated from 20 to 31 mm Hg and reduced from 30 to 4 mm Hg, respectively, after weaning from cardiopulmonary bypass. Intraoperative TEE and postoperative TTE showed normalized right ventricular function and septal motion. Anticoagulation therapy with warfarin was commenced from the third postoperative day. The inferior vena cava filter was also placed. One month after the surgery, CTPA and CT venography revealed no DVT and PTE. The patient was then discharged from the hospital without any complications. The patient provided informed consent for the publication of this case.

## 3 Discussion

Our patient was immobilized because of a pelvic bone fracture for 10 days before undergoing surgery under general anesthesia. The heart rate was mostly>100 beats/min, and the D-dimer test was positive. On the basis of the above information, the revised Geneva score^[8]^ and Wells score^[9]^ were 10 and 3, respectively, indicating an intermediate and moderate probability of having PTE (27.5–28.5% and 36.1–37.3%), respectively. However, anticoagulation therapy or intermittent pneumatic compression for the prevention of DVT or PTE was not performed because of the risk of bleeding^[10]^ and ischemic events distal to the pelvic bone fracture.^[11]^ Alternatively, CTPA and CT venography were performed to detect any PTE or DVT. Because of its non-invasiveness, availability, and high sensitivity (83%) and specificity (96%), CTPA is widely used in patients at high risk for PTE.^[12,13]^ CTPA combined with CT venography can diagnose DVT and PTE at the same time without using additional radiocontrast and exposure to excessive radiation.^[14]^ As the negative predictive value of CTPA was 89% in patients with a moderate probability (Wells score of 3) based on the results of a previous study,^[13]^ we believed that the negative findings of CTPA and CT venography almost excluded the preoperative development of PTE and DVT in our patient. Accordingly, preoperative echocardiography showed no evidence of PTE (visible embolus or right ventricular global dysfunction). As trauma produces fibrin, the usefulness of the levels of D-dimer is limited for the diagnosis of PTE in this case.^[15]^

Tachycardia occurs frequently in the early stage of PTE. Acute large or multiple PTE increases pulmonary vascular resistance, resulting in an increase in right ventricular afterload. The increased afterload causes right ventricular dysfunction and dilatation that displace the interventricular septum towards the left leading to a reduction in cardiac output and systemic arterial blood pressure, which is compensated for by tachycardia.^[16]^ Accordingly, sustained tachycardia and normal blood pressure were observed in clinical settings.^[17]^ In addition, the Wells score and revised Geneva score that were developed to predict the probability of PTE have heart rate (>100 and ≥ 95 beats/min, respectively) as one of the predictive variables.^[8,9]^ Particularly, the heart rate of ≥95 beats/min has the highest score among the variables included in the revised Geneva score.^[8]^ In our case, until the development of hemodynamic instability attributed to PTE, preoperative and intraoperative heart rates remained > 100 beats/min. Preoperative blood pressure was normal, and intraoperative blood pressure was maintained normal with continuous phenylephrine infusion. Unfortunately, all the perioperative efforts to control the rapid heart rate (administration of analgesics and beta-blockers and fluid management) were unsuccessful. Therefore, it was reasonable to assume that tachycardia was caused by PTE after excluding the candidate causes of tachycardia. However, the evidence of preoperative PTE and DVT was nearly absent on CTPA and CT venography findings. In addition, hypotension after anesthesia induction was easily treated with phenylephrine, and no other significant changes in vital signs were observed until the development of PTE during the surgery. Particularly, general anesthesia and surgery were additional causes of tachycardia (systemic vasodilatation, surgical stimulation, bleeding, preoperative dehydration, insensible loss, etc). Moreover, symptoms related to PTE were masked by general anesthesia. Therefore, it was very challenging to predict the intraoperative development of PTE in our patient.

Thus, the intraoperative use of TEE was not indicated for a prompt and accurate diagnosis of PTE in our case. However, it is very regrettable that we did not use TEE after anesthesia induction because PTE actually occurred in our patient. TEE is widely used to confirm the presence of pulmonary emboli and right ventricular dysfunction during surgery without affecting surgical performance.^[18–21]^ It is also helpful in the differential diagnosis of the causes of intraoperative hemodynamic instability, such as hypovolemia, pericardial tamponade, and severe hypokinesia in the left ventricular wall.^[22]^ In a report published in 2004, TEE performed by cardiac anesthesiologists could detect visible emboli immediately before surgery in only 26% of patients undergoing emergent pulmonary embolectomy for severe PTE.^[23]^ However, with advanced technology and increased levels of expertise, a recent study in 2014 reported that the evidence of emboli and the right ventricular strain was found in 87% and 92% of patients with intraoperative massive PTE, respectively.^[24]^ Although delayed, TEE monitoring was commenced after the occurrence of hemodynamic instability, and we found right ventricular dysfunction that indicated the development of PTE. However, pulmonary emboli could not be visualized presumably because of the lack of attending anesthesiologist's proficiency.^[25]^

At the beginning of CPR, we infused norepinephrine to increase arterial blood pressure and right ventricular contractility via strong α1 and modest β1 adrenergic effects, respectively. Another beneficial effect of norepinephrine is the restoration of coronary perfusion pressure gradients to the right ventricular subendocardium. In addition, fluid overloading was avoided to reduce right ventricular preload and systolic wall stress that worsen myocardial ischemia and ventricular interdependence. Although systemic administration of vasodilators, such as nitroglycerin or nitroprusside that release nitric oxide, can decrease pulmonary arterial and venous resistance, they may also worsen hypotension and systemic hypoperfusion because their action is not specific to the pulmonary vasculature.^[5]^ Hence, they were not used in our patient. As anticoagulation therapy is indicated for patients with normal blood pressure and right ventricular function,^[15]^ the therapy was not provided to our patient. The presence of hemodynamic instability indicated systemic thrombolysis or surgical embolectomy, and surgical embolectomy was performed because the patient had absolute contraindications to systemic thrombolysis (major trauma or surgery in previous 3 weeks and active bleeding).^[5]^

On the basis of our experience, negative CTPA and CT venography findings do not guarantee that intraoperative PTE does not occur in a patient with a moderate probability of having PTE. Although TEE was not used after anesthesia induction in our case, we strongly recommend the intraoperative use of TEE to investigate the causes of hemodynamic instability (including PTE) for patients with a moderate or high probability of having PTE. As tachycardia is an important predictive factor for PTE, anesthesiologists should consider the probability of intraoperative PTE occurrence if tachycardia is refractory to conventional treatments.

In conclusion, a comprehensive understanding of the risk factors and clinical features of PTE and monitoring and maintaining hemodynamics are mandatory for managing patients at risk of PTE.

## Author contributions

**Conceptualization:** Jong Hae Kim, Jung A Lim.

**Data curation:** Hyungseop Lim, Hyun Mi Kim, Jung A Lim.

**Writing – original draft:** Jong Hae Kim, Hyungseop Lim, Jung A Lim.

**Writing – review & editing:** Jong Hae Kim, Jung A Lim.
